# Disproportionately Large Communicating Fourth Ventricle: Pearls for Diagnosis and Management

**DOI:** 10.7759/cureus.3547

**Published:** 2018-11-05

**Authors:** Aswin Chari, Dimitrios Karponis, Claudia L Craven, Akbar A Khan, Lewis Thorne

**Affiliations:** 1 Neurosurgery, Imperial College London, London, GBR; 2 Orthopaedics, Imperial College London, London, GBR; 3 Neurosurgery, National Hospital for Neurology and Neurosurgery, London, GBR; 4 Neurosurgery, Queen Elizabeth University Hospital, Glasgow, GBR

**Keywords:** communicating hydrocephalus, fourth ventricle, negative pressure shunting, ventriculopleural shunt

## Abstract

Introduction

Disproportionately large communicating fourth ventricle (DLCFV) is an unusual presentation of communicating hydrocephalus, in which patients with hydrocephalus have a disproportionately enlarged fourth ventricle in the absence of obstructive pathology. We present six cases of DLCFV which, to date, is the largest series of this relatively rare condition. We highlight the significance of diagnosis and its differentiation from trapped fourth ventricle (TFV) and discuss the nuances for optimal management of DLCFV.

Methods

Retrospective case series of consecutive patients with DLCFV, managed by the senior author (LT) over a 10-year period.

Results

Six cases were identified, five of whom had previous posterior fossa surgery and one with previous encephalitis. All patients presented with cerebellar signs, the initial group had unsuccessful initial management with typical cerebrospinal fluid (CSF) diversion. Consistent symptom resolution was achieved by the application of negative CSF pressures via external ventricular drainage (EVD), maintained with subsequent ventriculopleural shunt (VPL), valveless lumbopleural shunt (LPS) or valveless ventriculoperitoneal shunt (VPS), or proceeding directly to a low-pressure system.

Conclusions

DLCFV is a diagnosis characterised by cerebellar dysfunction, with or without cranial nerve palsies, often in the setting of previous posterior fossa pathology. Optimal management relies on knowledge of this unique diagnostic entity, and use of an EVD at negative pressures to confirm symptomatic and radiological improvement prior to definitive treatment.

## Introduction

Communicating hydrocephalus can occur as a result of a number of different pathological processes and is usually characterised by proportionate enlargement of the lateral, third and fourth ventricles [1]. A subgroup of patients with communicating hydrocephalus have a fourth ventricle that is enlarged to a greater extent than the lateral and third ventricles, in the absence of an obstructive pathology. This phenomenon, termed disproportionately large communicating fourth ventricle (DLCFV) was first documented in 1978. Subsequently a limited number of case reports have been published, almost exclusively in the Japanese literature [2-15].

The majority of the aforementioned patients present with cerebellar signs (ataxia and nystagmus) and cranial nerve palsies, and often without the traditional signs and symptoms of raised intracranial pressure (headache, nausea, vomiting, altered consciousness and papilloedema). Treatment is often difficult and patients either undergo multiple shunt revisions or require additional procedures prior to symptom resolution [2-5, 7, 8, 10, 11, 13].

Effective management depends on early identification of the condition. It is easy to mistake this entity for a more common communicating hydrocephalus or an isolated fourth ventricle. This is likely to lead to treatment failure or an unnecessary attempt to cannulate the fourth ventricle. Our case series demonstrates that this is a distinct entity characterised by a low-pressure state in patients who are likely to have altered compliance in the posterior fossa, either through disease or previous intervention.

We present a retrospective case series of DLCFV, outlining the aetiology, clinical and radiological findings and outcomes for these patients. We present a novel treatment paradigm for this condition, which results in consistent radiological and symptomatic improvement, without a need for multiple surgical interventions and shunt revisions.

## Materials and methods

**Figure 1:**
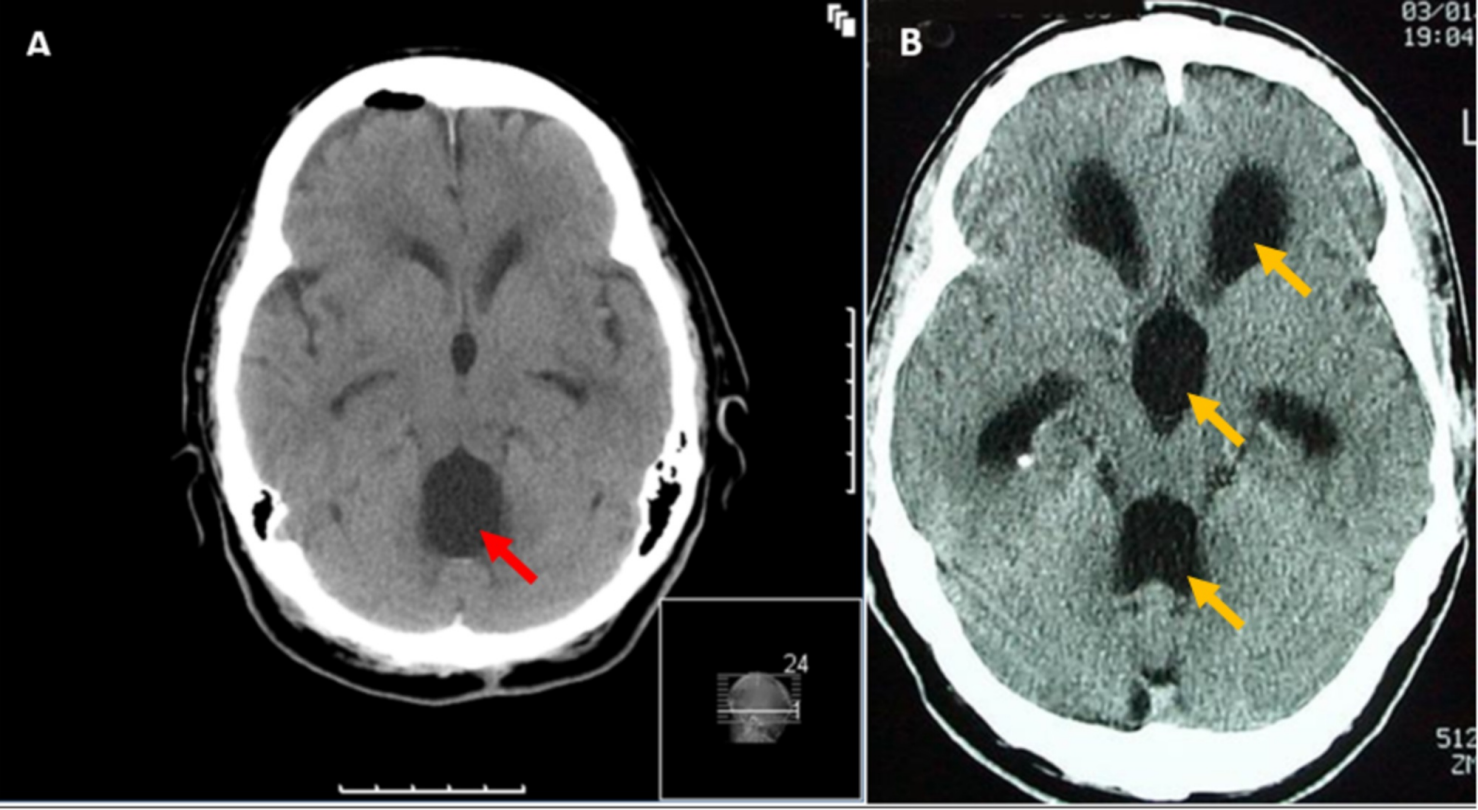
Typical CT image of DLCFV (A) compared with the proportionate enlargement of all ventricles more often seen in a communicating hydrocephalus (B). Note that in DLCFV only the fourth ventricle is disproportionately enlarged (as shown by the red arrow), compared to a universal enlargement (as shown by the yellow arrows). CT: Computed tomography; DLCFV: Disproportionately large communicating fourth ventricle.

Consecutive cases of DLCFV managed by the senior author (LT) over the last 10 years (2006-2015) were retrospectively identified (Figure 1). Case notes and imaging were retrospectively examined to identify the aetiology, clinical and radiological presentation, treatment and outcomes for these patients.

## Results

Clinical characteristics

Six cases were identified. The patient characteristics are summarised in Table 1. Five patients had posterior fossa surgery with past interventions prior to presentation with DLCFV and one patient had previous encephalitis. There were no cases of idiopathic DLCFV. The patient presentation was characterised by cerebellar signs (ataxia, nystagmus and nausea). After presentation, four patients were initially treated with ventriculoperitoneal shunt (VPS) (programmable system incorporating an anti-siphon device) or shunt revision (SR); none of these patients experienced symptomatic improvement and showed persistent fourth ventriculomegaly.

**Table 1:** Presenting aetiology, symptoms and treatment of six patients in this series with DLCFV. vVPeS: valveless ventriculoperitoneal shunt; DLCFV: Disproportionately large communicating fourth ventricle; EVD: External ventricular drainage; HIV: Human immunodeficiency virus; PICA: Posterior inferior cerebellar artery.

Patient	Aetiology	Clinical Presentation	Radiological Presentation	Initial Treatment	Subsequent Temporary Treatment	Definitive Treatment
1	Post fossa craniectomy for resection of recurrent craniocervical junction meningioma	Eight months post resection – Ataxia, somnolence	DLCFV	VPeS insertion and revision following persistent ventriculomegaly	EVD resulted in ventricular collapse and when EVD set below 0 cm H_2_O	vVPeS
2	Post fossa craniectomy for clipping of PICA aneurysm + ventriculoperitoneal shunt insertion	Rebleed and shunt producing acute shunt failure – Headache, ataxia, nystagmus	DLCFV	VPeS revision x2 following persistent ventriculomegaly	EVD with ventricular collapse and symptomatic improvement when EVD set below 0 cm H_2_O	Ventriculopleural shunt incorporating fixed low-pressure differential pressure valve
3	HIV encephalitis	Ataxia, somnolence, facial palsies, nystagmus	DLCFV	VPeS insertion and revision x2 following persistent ventriculomegaly of fourth ventricle	EVD with ventricular collapse and symptomatic improvement resulting from progressive lowering of EVD pressure, final setting - 20 cm H_2_O	vVPeS
4	Post fossa craniectomy for vestibular schwannoma	Presented three months post procedure with headache, nausea and vomiting	DLCFV	VPeS insertion and revision with persistent ventriculomegaly of fourth ventricle		Programmable lumber peritoneal shunt
5	Foramen magnum decompression for Chiari I malformation	Ataxia, nystagmus	DLCFV	EVD with ventricular collapse and symptomatic improvement resulting from progressive lowering of EVD pressure, final setting - 10 cm H_2_O		Ventriculopleural shunt incorporating fixed low-pressure differential pressure valve
6	Vestibular schwannoma treated with stereotactic radiosurgery	Ataxia and nystagmus	DLCFV	Lumbar drain with ventricular collapse and symptomatic improvement resulting from progressive lowering pressure, final setting - 10 cm H2O		Programmable lumber peritoneal shunt

Intervention

In four patients, temporary treatment with an external ventricular drain (EVD) or lumbar drain (LD) was employed. Symptomatic improvement and corresponding resolution of fourth ventricular enlargement occurred only when the drain was set below 0 cm H_2_O; in one case, this was as low as -20 cm H_2_O.

Definitive treatment, therefore, sought to establish a low-pressure system capable of maintaining negative pressures. This was in the form of a valveless ventriculoperitoneal shunt (vVPS; two patients), a ventriculopleural shunt (two patients) or a lumboperitoneal shunt (LPS; two patients).

Clinical and radiological outcome

All patients improved symptomatically following low-pressure cerebrospinal fluid (CSF) diversion, with complete resolution of ataxia, nystagmus and somnolence. No patients required further revision or intervention after low-pressure CSF diversion.

**Figure 2:**
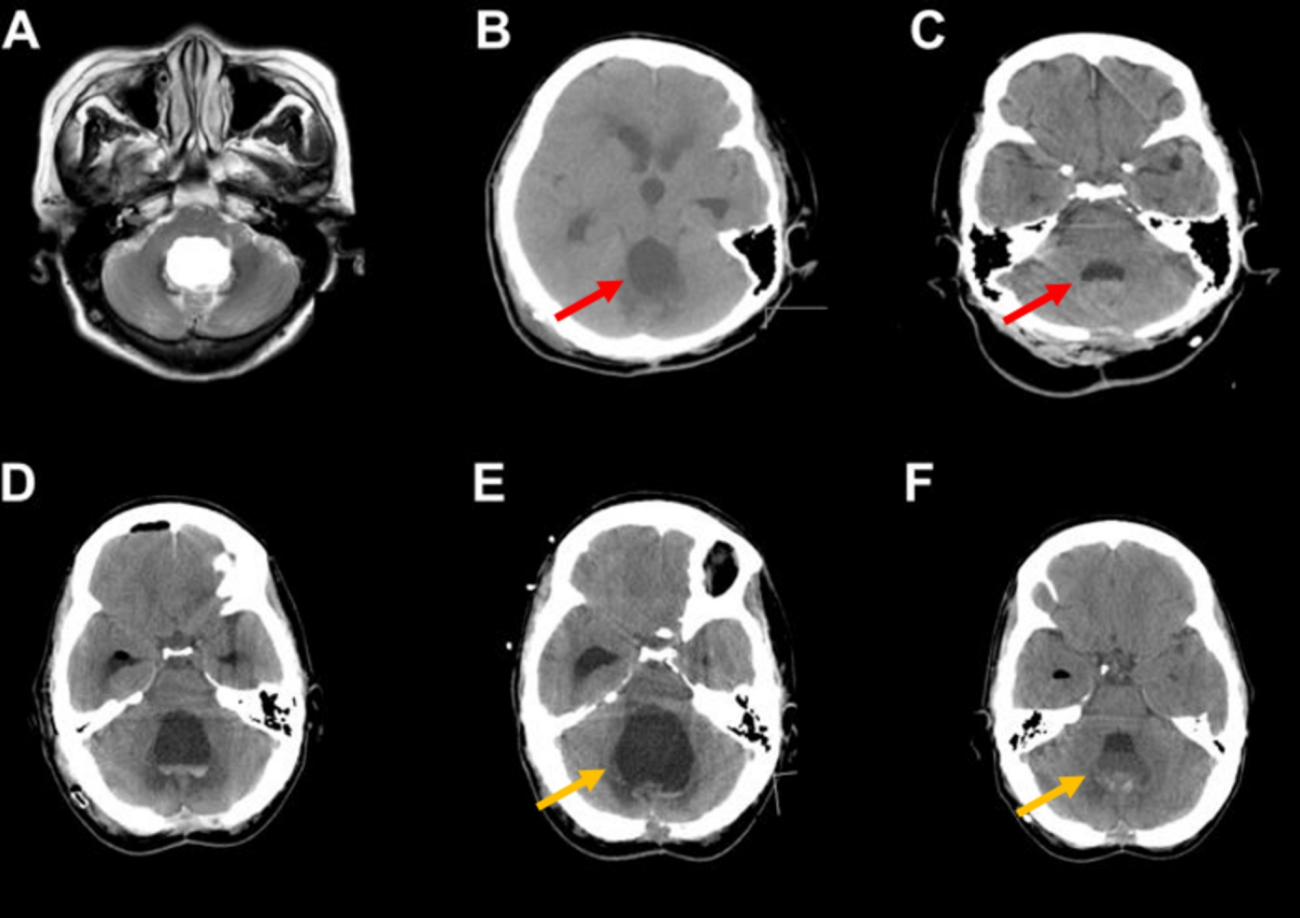
Example of two patients with DLCFV at the time of presentation (A and D), after ventriculoperitoneal shunting (B and E) and after definitive treatment with a low-pressure system; valveless ventriculoperitoneal shunt (C) or ventriculopleural shunt (F). Note that both patient A (red) and D (yellow) show markedly reduced size of their fourth ventricle following low pressure shunting (C and F), unlike with traditional ventriculoperitoneal shunting (B and E). DLCFV: Disproportionately large communicating fourth ventricle

Figure 2 shows examples of fourth ventricular size at presentation in two patients (A and D), after initial VPS (B and E) and following subsequent treatment with vVPS (C) or lumboperitoneal shunt (F).

## Discussion

We present six cases of DLCFV which, to date, is the largest series of this relatively rare condition. Optimal management was achieved through negative pressure CSF diversion. We highlight the potential pitfalls, salient points and pearls in the management of DLCFV.

Presentation of DLCFV

In our case series, DLCFV occurred in patients with previous posterior fossa pathology and there were no ‘idiopathic’ cases observed. The preceding posterior fossa disease or intervention suggests that the aetiology is likely to involve an inflammatory process resulting in altered compliance of the fourth ventricle. All patients with DLCFV characteristically present with cerebellar symptoms of nausea, ataxia and often had nystagmus. DLCFV was often associated with particularly patulous foramen of Lushka on magnetic resonance imaging (MRI), and importantly, all patients had communicating hydrocephalus with a patent aqueduct of Sylvius.

DLCFV versus the trapped fourth ventricle

Clinically, DLCFV and syndrome of the isolated (or trapped) fourth ventricle (TFV) present in a similar fashion. Distinguishing DLCFV from TFV is a critically important. TFV is a form of non-communicating hydrocephalus for which the treatment is very different; it may require a separate shunt into the fourth ventricle and/or additional open/endoscopic procedures to ensure the fourth ventricle communicates with either the third ventricle and/or the subarachnoid space [16]. If applied to patients with TFV (or other non-communicating forms of hydrocephalus), the low-pressure CSF drainage described in this series could result in severe differential transtentorial pressures, leading to lower cranial nerve palsies and ataxia (referred to a Transtentorial Distortion Syndrome). MRI with CSF flow studies can confirm if there is flow within in aqueduct for cases that are difficult to differentiate.

Management of DLCFV

If there is doubt over the initial diagnosis of DLCFV, confirmatory testing may be performed through a temporary EVD. Symptomatic and radiological improvement should be experienced when the drain is set at progressively lower pressures; sometimes as low as -20 cm H_2_O. An initial very low pressure setting may encourage return of the ventricles to normal size, required due to pressure-volume hysteresis curve [17]. In addition to establishing the pressures required for symptomatic and radiological improvement, temporary external drainage is also useful for differentiating between DLCFV and the syndrome of TFV [16], and in providing reassurance that there is patent CSF drainage when the ventricles do not come down.

In our experience, DLCFV does not respond to VPS. This may be because our initial shunt management was with a programmable system incorporating an anti-siphon device that would have been unable to generate the low CSF pressure required to resolve the condition, as demonstrated by progressive lowering of EVD drainage pressure. Successful management required the establishment of a low-pressure system (often negative pressure system), for example via a ventriculopleural shunt that is able to generate negative pressures during inspiration.

Finally, we caution against attributing the initial lack of response to shunting to shunt failure. Such patients are at risk of being exposed to multiple and unnecessary VPS revisions if early low-pressure CSF diversion is not considered.

Literature review

Since the initial reports by Zimmerman et al. from the USA [15], DLCFV has almost exclusively been reported in the Japanese literature. A literature search revealed a total of 22 cases from 14 manuscripts. Those with sufficient English language reporting are summarised in Table 2. The patients presented in the literature seem to have a number of characteristics in common with those presented in the current series. Firstly, a number of them have had previous neurosurgical pathology, either in the posterior fossa or due to subarachnoid and intraventricular haemorrhage. Secondly, the clinical presentation is consistent with the features of DLCFV, namely cerebellar signs in addition to the traditional signs and symptoms of hydrocephalus.

**Table 2:** Presenting aetiology, symptoms and treatment of nine patients reported in the literature with DLCFV. DLCFV: Disproportionately large communicating fourth ventricle; SAH: Subarachnoid hemorrhage; VPS: Ventriculoperitoneal shunt; EVD: External ventricular drainage; LPS: Lumbopleural shunt; AVM: Arteriovenous malformation; ICH: Intracerebral haemorrhage; IVH: Intraventricular haemorrhage; MCA: Middle cerebral artery.

Reference	Case	Aetiology	Clinical Presentation	Radiological Presentation	Initial Treatment	Definitive Treatment
Yamashita et al., 2012 [2]	44F	SAH due to right vertebral artery dissecting aneurysm + VPS	Presented with headache, nausea, vomiting and truncal ataxia	Diffuse ventricular enlargement with valve opening pressure at 115 mm H_2_O and DLCFV on reduction of opening pressure to 45 mm H_2_O + C1-C7 Syringomyelia + C6-T2 anterior intradural arachnoid cyst	VPS	Additional fourth ventricular-peritoneal shunt with independent pressure control
Katano et al., 2012 [3]	6wk F	Meningomyelocele repair + VPS	Poor agility, grim-face, occasional choking on milk and downward gaze.	DLCFV + Cervical syringomyelia	-	Reduction of Strata NSC valve setting from 1.5 to 1.0
Hagihara and Sakata 2007 [4]	13M	Unknown	Headache, nausea, gait disturbance, truncal ataxia, nystagmus and incontinence	DLCFV + Whole spine syringomyelia	-	VPS
Shose et al., 1991 [8]	39M	AVM resection (?location) + VPS	Altered consciousness, diplopia, rotatory nystagmus, drowsiness, truncal ataxia and Parinaud’s sign	DLCFV	EVD with ventricular collapse and symptomatic improvement (? level)	VPS
Toriyama et al., 1991 [10]	27M	Traumatic ICH + IVH + VPS	Nausea, vomiting, altered consciousness and nystagmus	DLCFV	-	Fourth ventricular-peritoneal shunt
Okabe et al., 1990 [11]	21M	Multiple IVH from parietal AVM + VPS	Altered consciousness, bilateral exotropia	DLCFV	.-	Revision of VPS
Okabe et al., 1990 [11]	66F	SAH + IVH due to right MCA aneurysm	Altered consciousness, bilateral exotropia	DLCFV	^-^	No improvement despite VPS + LPS + VPS revision. Patient died
Matsumoto et al., 1983 [13]	24M	Post lumbar discectomy	Nystagmus, Parinaud’s sign, truncal ataxia and symptoms typical of hydrocephalus	DLCFV	-	VPS
Matsumoto et al., 1983 [13]	22F	SAH	Nystagmus, Parinaud’s sign, truncal ataxia and symptoms typical of hydrocephalus	DLCFV	-	VPS
Zimmerman et al., 1978 [15]	43M	Unknown	Headaches, gait ataxia and nystagmus	DLCFV	-	VPS
Zimmerman et al., 1978 [15]	10F	Intrauterine rubella and congenital hydrocephalus	Behavioural abnormalities and gait/balance problems	DLCFV	VPS	Fourth ventricular-peritoneal shunt

Management of the patients in the literature was varied, with most patients improving with standard VPS. Few studies reported the type of shunt and valve used and therefore, the pressure dynamics of those systems are unknown. It is important to consider that a standard differential pressure valve without an antisiphon device will generate negative pressures in the standing patient that might be sufficient to manage a proportion of these patients.

The use of additional shunts, such as fourth ventricular-peritoneal shunts, creates increasingly complex systems with increased risk of shunt failure and need for further operative interventions. In addition, the literature is limited by a heterogeneous definition of DLCFV; some authors report the phenomenon of DLCFV in cases where there is clear fourth ventricular outlet obstruction with a patent aqueduct and no ‘disproportionate enlargement’ of the fourth ventricle, which, in our opinion, is a separate entity of obstructive hydrocephalus and should be managed differently, such as with endoscopic third ventriculostomy [5, 14]. The term DLCFV should be limited to patients with a patent CSF circulatory system (flow through the aqueduct and fourth ventricular outlets) and therefore a truly communicating hydrocephalus.

Study strengths and limitations

Whilst this study is limited by the small numbers, this is a rare condition in adults and this series of six patients is the largest on DCLFV to date. The complete and persistent resolution of symptoms after negative pressure CSF diversion was a consistent finding in a heterogeneous group of patients.

## Conclusions

This case series of six cases of DLCFV illustrates the importance of awareness of this condition in directing definitive management. Traditional VPS, even with a valve set at low pressures, may not result in symptomatic improvement and therefore, a systematic approach using temporary CSF diversion via an EVD to assess the pressures required for fourth ventricular collapse and corresponding symptomatic improvement prior to definitive treatment with either a vVPS, an LPS or ventriculopleural shunt seems to be the most pragmatic approach to these patients.
